# Ultrasound Imaging Modalities in the Evaluation of the Dog’s Stifle Joint

**DOI:** 10.3390/vetsci12080734

**Published:** 2025-08-04

**Authors:** Anargyros T. Karatrantos, Aikaterini I. Sideri, Pagona G. Gouletsou, Christina G. Bektsi, Mariana S. Barbagianni

**Affiliations:** Faculty of Veterinary Medicine, University of Thessaly, Trikallon 224, 43100 Karditsa, Greece; akaratrantos@uth.gr (A.T.K.); ksideri@uth.gr (A.I.S.); cbektsi@uth.gr (C.G.B.)

**Keywords:** ultrasonography, Doppler, CEUS, contrast-enhanced examination, elastography, knee, B-mode, ligaments, tendons, canine

## Abstract

This review presents a comprehensive overview of various ultrasound imaging techniques employed in the evaluation of the canine knee joint. It critically analyzes studies conducted on both human and animal subjects, with a focus on the diagnostic accuracy of B-mode ultrasound, Doppler examination, contrast-enhanced ultrasound, and elastography in both normal and pathological conditions. The review underscores the necessity of strict adherence to the protocols of each ultrasound modality and emphasizes the importance of a thorough understanding of the anatomical region to achieve optimal outcomes. The findings suggest that these ultrasound techniques can significantly enhance the diagnostic process, providing valuable insights into anatomy, size, blood supply, and tissue elasticity. Additionally, in cases where advanced imaging modalities such as computed tomography (CT) or magnetic resonance imaging (MRI) are cost-prohibitive or less accessible, ultrasound serves as a reliable alternative, delivering high diagnostic accuracy and critical information regarding mechanical changes in the joint and neovascularization.

## Simple Summary

This review aims to describe the various ultrasound techniques used for evaluating the stifle joint in dogs, emphasizing the specific advantages and disadvantages of each method. The ultrasound examinations that can be performed include B-mode, Doppler examination, contrast-enhanced examination, and elastography. Each ultrasound modality provides unique information that can enhance the overall diagnostic approach. It is important to note that ultrasound is not intended to replace other imaging modalities, such as computed tomography or magnetic resonance imaging. However, in clinical practice, factors such as cost and availability of computed tomography or magnetic resonance imaging may limit their use, making ultrasound examination a more feasible option in certain situations. Therefore, the examiner must possess a comprehensive understanding of the anatomical area and carefully select the appropriate methodology based on the suspected pathological condition and the specific diagnostic information required.

## 1. Introduction

Musculoskeletal ultrasound is a growing field in human and veterinary medicine [1,2]. This review utilized a narrative approach. The literature search was conducted in July 2024 and updated in May 2025 by three authors who independently screened the search results across three databases: MEDLINE via PubMed, Web of Science, and Google Scholar. The keywords used included “ultrasonography”, “dog’s stifle joint”, “cranial cruciate ligament”, “meniscus”, “patellar ligament”, “ultrasonography techniques”, and their synonyms in various combinations. To be included, studies had to describe ultrasound examinations or anatomical structures related to the knee. There were no restrictions on the year of publication or the publication type. However, articles that focused on referrals for ultrasound examinations, therapeutic ultrasound, or those describing ultrasound without producing an image were excluded. Additionally, articles not published in English or German were also excluded. Furthermore, relevant textbooks and anatomy books were also utilized in the search. This background sets the stage for understanding the contribution and evolution of ultrasonographic techniques in knee joint evaluation.

The first descriptions of ultrasonographic investigation of the human knee joint date from the late 1980s [1,2]. Since 2001, there has been a specific protocol for the ultrasonographic human knee examination [3]. In the 1990s, researchers described the ultrasonographic examination of the equine stifle [4,5]. Ultrasonographic evaluation of the canine stifle was published by Reed et al. in 1995, stating that ultrasound can be used to image the normal anatomy of this joint, and that its ultrasonographic appearance has similarities with the equine and human stifle [6]. In dairy cows, ultrasonography is employed to identify abnormalities in bones, tendons, ligaments, and synovial structures, particularly in cases of foot disorders [7,8].

The stifle joint is a common site of lameness in dogs, making the etiological diagnosis of the pathological condition of great importance. The most common disorder of the canine stifle joint is the cranial cruciate ligament (CCL) rupture [9]. The etiopathogenesis of CCL rupture is multifactorial and remains controversial, involving interactions among genetic predisposition, biological processes, and mechanical stresses. Gradual degeneration of the ligament leads to partial rupture, which may eventually result in complete rupture, joint instability, and progressive osteoarthritis (OA) [10]. However, diagnosing it clinically can be difficult in some dogs [11]. Meniscal injuries, typically associated with CCL rupture, are also a common cause of morbidity. Their diagnosis is challenging, as it cannot be confirmed through clinical examination. Notably, persistent lameness following stabilization surgery of CCL rupture is often attributed either to undetected meniscal injury at the time of surgery or to late-onset meniscal injury [10]. Under these circumstances, when metallic implants are present, the diagnostic utility of advanced imaging modalities such as MRI may be limited [10]. Other frequently observed pathological conditions of the stifle joint, including patellar luxation [12] and osteochondrosis in the medial or lateral femoral condyle [13,14], pose significant challenges for effective diagnostic imaging. These difficulties arise from the small joint structures, the limited joint space, and the complex anatomy of the area. The overlapping of different tissue types further complicates successful imaging when relying on a single imaging modality [12].

Ultrasound evaluation offers several advantages compared to other imaging modalities, including greater availability, quicker procedures, ease of repeat examinations, and lower costs [15]. It is a practical, dynamic, and interactive assessment method [16] that provides high-resolution imaging. In fact, ultrasound machines currently in use deliver remarkably detailed images of the musculoskeletal system, providing resolution sometimes similar to magnetic resonance imaging (MRI) [17]. Like other imaging modalities, musculoskeletal ultrasound allows for the use of the contralateral limb for comparison, enabling an effective evaluation of the symmetry of tendinous structures [18]. However, this comparison can be challenging in cases of bilateral orthopedic disease, as ultrasonographic findings often differ between limbs. In such cases, assessing asymmetrical tendon findings between limbs can be particularly informative [18]. Another advantage is that diagnostic ultrasound can be performed on awake or lightly sedated patients, and it rarely requires the use of general anesthesia. Moreover, it is a dynamic study and focuses the examination on the symptomatic area [19]. Finally, ultrasound offers real-time guidance for interventional procedures, facilitating safe and precise biopsy needle placement [20].

The effectiveness of ultrasound examination in small animal practice heavily depends on both the quality of the ultrasound equipment and the examiner’s expertise, as well as their understanding of the anatomy of the areas being examined [21]. The examiner should also be able to identify and address various artifacts during ultrasonographic examination [22].

The ultrasonographic techniques that are applied for the assessment of the stifle joint are the B-mode, Doppler (color, pulsed wave), contrast-enhanced ultrasound (CEUS), and elastography techniques. Doppler ultrasonography is an accurate, non-invasive technique used to assess blood flow characteristics [23]. Doppler ultrasonography has been applied to monitor vascular blood flow dynamics in the feet of healthy horses or horses affected by laminitis and septic pododermatitis [24,25]. Moreover, it is recommended for the estimation of neovascularization during fracture healing in dogs [26] and arthritis [27,28].

CEUS contributes to the assessment of microcirculation in muscle pathology, tendinopathy, fracture nonunions, arthritis, and tumors [29,30,31].

Elastography is an effective, quick, and safe ultrasound imaging method that evaluates tissue stiffness and is applied for the evaluation of dogs’ musculoskeletal tissues [32,33,34,35,36,37]. Two different types of elastography are usually used, strain elastography (SE) and shear-wave elastography (SWE). SE is a subjective method that relies more on the operator than SWE, as it requires the manual compression of the tissues via the transducer [38,39,40]. According to the human literature, SWE provides more objective and quantitative data than SE [40]. Elastography is highly valuable in human medicine for the assessment of Achilles tendon and patellar ligament tendinopathies [41,42]. In veterinary medicine, it is equally important for evaluating tendon and ligament injuries in horses, facilitating the characterization of both the chronicity and severity of lesions [43].

## 2. Methodology of the Examination of the Stifle Joint

Musculoskeletal ultrasound involves the use of high-frequency sound waves [20] to produce detailed anatomic images of tendons, nerves, ligaments, joint capsules, and muscles [20] and to identify both bony and soft tissue injuries [18,44,45].

Generally, effective imaging requires positioning the transducer so that the beam is perpendicular (at a 90° angle) to the structure being examined [46]. The cranial aspect of the stifle should be assessed with the joint flexed at a 90° angle [6]. For the evaluation of the lateral joint space, the transducer is placed laterally to produce a sagittal image [47]. The menisci are usually assessed in three longitudinal planes: cranio-abaxial, abaxial, and caudo-abaxial. The cruciate ligaments should be examined in fully flexed joints [6]. Finally, the patellar ligament should be imaged longitudinally and transversely [4,5,47,48]. The popliteal artery should be examined in the caudal region of the stifle using sagittal and transverse images, with the knee positioned in slight flexion [47].

The bibliography proposes the lateral recumbency position of the dog during the examination, with the stifle joint to be examined facing upward, without the need for sedation [48]. The limb should be clipped from the distal third of the femur to a few centimeters below the tibial tuberosity. The skin should be cleansed with 70% ethanol, and a generous amount of ultrasound coupling gel should be applied [48]. Due to the shape and the size of the joint, coupling of the transducer is difficult [21]. Proper technique necessitates ensuring adequate skin contact and choosing the appropriate transducer size for the specific situation [44]. For joint ultrasonography in dogs, it is important to utilize an ultrasound machine with a high-resolution probe operating at 8–16 MHz [48,49]. The ultrasonographer should be adept at positioning the stifle correctly and placing the probe at the right angle to capture adequate images [49]. Additionally, extension and flexion of the stifle would enhance the visibility of internal structures [49].

Pulsed-wave (PW) Doppler ultrasound is performed on a longitudinal section of the vessel [50]. In color Doppler ultrasound scanning, various settings are often employed, such as the color box, color gain, color velocity scale, and inversion. The color box is a square area that shows all color Doppler information, and its size and depth are crucial for the imaging quality [51]. It is advisable to keep the box as small as possible, and the “steer” button should be aligned with the arterial axis. Color gain is set at a level to capture the color within the arterial lumen [51]. Key parameters in a PW examination include the following:

- Angle of insonation: The angle between the direction of blood flow and the ultrasound waves should be less than 60°.
- Doppler gate: The cursor is positioned in the center of the vessel, occupying half to two-thirds of its diameter [52].
- Doppler angle: Should be set between 45° and 60° [53].
- Pulse repetition frequency: Should be adjusted based on the blood flow velocity.
- PW Gain: Should be set to ensure optimal contrast between the Doppler waveform and the background [54].

CEUS utilizes an intravascular contrast agent, SonoVue ^®^ (Bracco, Milan, Italy), which contains sulfur hexafluoride [29]. The quantitative parameters of CEUS are determined by observing the dynamic changes in the contrast agent over time within a fixed region of interest (ROI). The time-intensity curve (TIC) of the contrast agent’s perfusion typically exhibits an initial exponential rise, reaches a peak, and then gradually declines [31,55]. The key parameters that should be assessed include the following:

- The time interval from contrast injection to its arrival at the region of interest and subsequent detection by ultrasound.
- The maximum intensity of the contrast agent, known as Peak Enhancement (PE), and the time taken to reach PE.
- The regional blood volume area.
- The maximum value of the enhancement curve, referred to as the Wash-In Rate.
- The time from contrast agent injection until PE.
- The duration during which the intensity exceeds the mean value.

Regarding the elastography procedure, sedation may be crucial, as it helps reduce movement from both the patient and the ultrasonographer [56]. The longitudinal sections were suggested for the elastography examination of the stifle to ensure proper contact between the probe and the examined tissue. Moreover, dogs were proposed to be examined in a standing position [34]. Piccionello et al. [33] recommended performing patellar ligament elastosonography with the stifle in full passive flexion; however, they did not evaluate the elastosonography readings for any other stifle position to detect any differences. The literature in humans suggests that the middle of the patellar tendon is the optimal site for elastography due to its capacity to yield the most consistent measurements [57]. Multiple SE canine studies have focused exclusively on the middle of the patellar tendon [33,34,37]. Additionally, a study by Embriano et al. [56] advocates for SWE measurements to be taken from the proximal or middle sections of the patellar tendon when the stifle is extended to 150° or more.

## 3. Imaging of Normal and Pathological Musculoskeletal Structure of the Stifle

### 3.1. Imaging of Normal Musculoskeletal Structure

In musculoskeletal ultrasonography, tissues are evaluated based on their echogenicity (isoechoic, hypoechoic, anechoic, hyperechoic), echotexture (the internal echo pattern), degree of anisotropy, compressibility or elasticity, and the presence or absence of blood flow as detected by Doppler examination [20].

#### 3.1.1. Muscle

Healthy muscle appears with a hyperechoic surface, while the muscle belly itself is hypoechoic [21,58]. In longitudinal imaging, the fine hyperechoic striations indicate the connective tissue between muscle fascicles. Conversely, in transverse imaging, multifocal hyperechogenicities are visible [59].

#### 3.1.2. Tendon and Ligament

Healthy tendons and ligaments appear as fine, thin structures with hyperechoic parallel fibers, typically visualized near the musculotendinous junction [60,61]. A small amount of hypoechoic fluid may be present superficially in the tendons and within the tendon sheaths [18]. A normal ligament resembles a tendon, appearing as a hyperechoic linear structure with a more compact fibrillar echotexture due to its connective tissue composition [62]. When viewed longitudinally, tendons exhibit a fibrillar pattern, while a “broom-end” pattern is seen in transverse images [63,64,65]. Evaluating ligaments transversely can be challenging due to their thin structure [66]. A key distinguishing feature is that ligaments attach to bone, so tracing the structure to its bony insertion is essential for differentiating it from a tendon [16,45,64].

#### 3.1.3. Bone

Bone is generally imaged as a hyperechoic continuous line with a smooth surface and demonstrates distal acoustic shadowing [18,67]. Ultrasound imaging of bone is restricted to assessing the superficial features of the visible bone structures [46,62].

#### 3.1.4. Stifle Joint

The recognizable structures of the stifle joint consist of the patellar ligament and tendon, the cranial joint space, which includes the infrapatellar fat pad, synovium, and CCL, along with both the medial and lateral menisci [6,12,47]. On ultrasound, a normal joint shows a uniform bone profile, consistent echogenicity of the periarticular soft tissues, and may have a small amount of fluid present in the joint recess or bursae [58]. Hyaline cartilage is observed as a clearly defined anechoic or uniformly hypoechoic band positioned between the chondrosynovial and osteochondral margins [58]. The entire cartilage appears as a regular, non-echogenic line situated between the hyperechoic interface of the hyaline cartilage layer and the joint capsule [47,68,69]. Regarding the elasticity of the joint, a study by Diogo et al. (2020) using acoustic radiation force impulse (ARFI) elastographic examination in the stifle joint of healthy dogs found that elasticity decreased with age and that female structures exhibited greater stiffness compared to males [36].

- Cranial Cruciate Ligament (CCL)

The assessment of the CCL using ultrasound is often difficult due to its orientation [6,48,70]. Typically, the distal portion of the ligament appears hypoechoic in comparison to the patellar ligament and is surrounded by the echogenic infrapatellar fat [6].

- Patellar ligament

**Figure 1:**
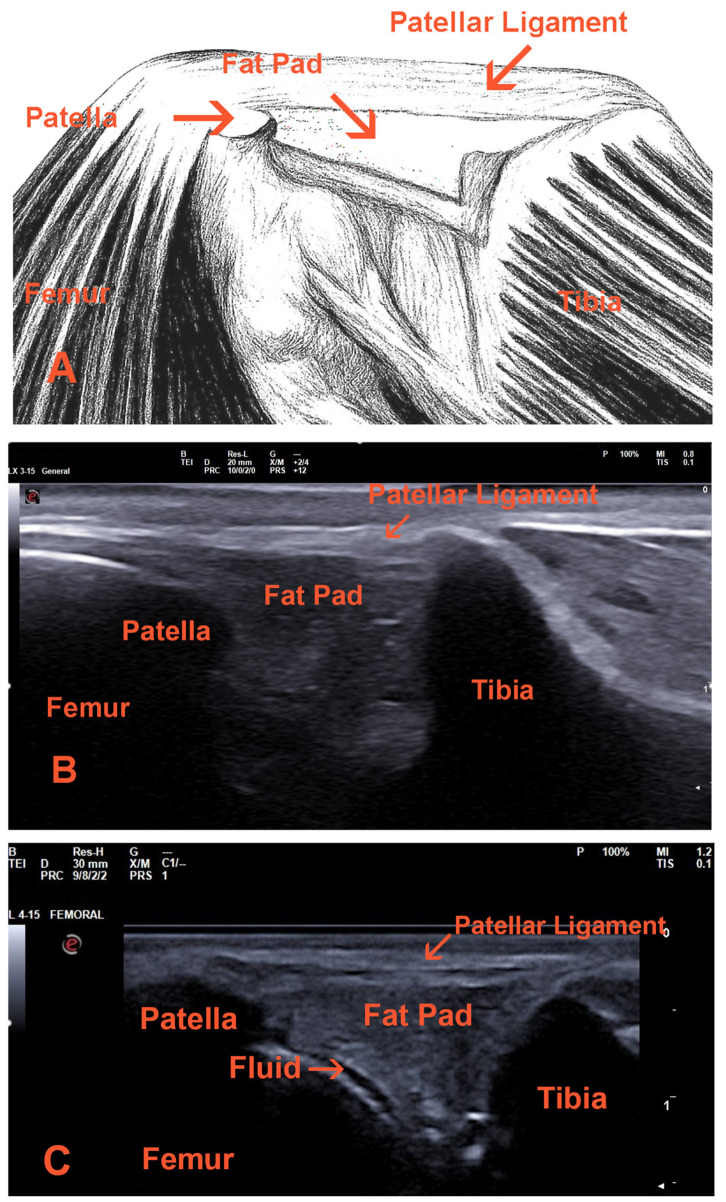
(**A**) Schematic illustration of the dog’s stifle joint, depicting the lateral aspect. (**B**) Ultrasonographic image of a normal stifle joint in a dog, captured from the lateral view. (**C**) Ultrasonographic image of the stifle joint in a dog with patellar luxation, with an arrow indicating the presence of anechoic fluid, which suggests effusion. The images were obtained using an Esaote MyLab™ Sigma ultrasound machine with a linear probe (L 4-15) (Esaote, Genoa, Italy) operating at a frequency of 12 MHz and a depth of 2 cm. (Ultrasound images: A. Karatrantos.)

The normal canine patellar ligament is visualized as a thin, hyperechoic structure with a homogeneous, parallel fiber pattern, encased in a thin, echogenic periligamentous sheath [6,47] (Figure 1). Regarding elastography, the normal canine patellar ligament displays a very soft elastogram, like in humans [33,37,71,72].

- Other tendons and ligaments

Ultrasonographic visualization of the superficial tendons, such as the quadriceps and long digital extensor, as well as collateral ligaments of the stifle, is feasible [48]. However, visualizing the caudal cruciate ligament is very challenging to impossible due to the significant muscle mass in that area [6,48].

- Meniscus

Visualizing the entire meniscus ultrasonographically can also be quite challenging [6,47,49,73]. The menisci are characterized by diffuse echogenicity and a moderately fine echotexture [49]. They are wedge-shaped in cross-section structures positioned along the medial and lateral aspects of the joint. Due to their “C” shape, it is essential to carefully examine the cranial, central, and caudal portions of both menisci [6,12,47].

#### 3.1.5. Peripheral Nerve

Generally, in longitudinal ultrasound views, peripheral nerves appear as parallel hyperechoic lines separated by hypoechoic areas. In transverse views, these nerves exhibit multiple punctate echogenicities within a well-defined, ovoid nerve sheath [62]. The ischiatic nerve branches extend into the knee region in dogs [74]. The normal sciatic nerve appears as a hypoechoic tubular structure, containing parallel echogenic linear structures within. It is also defined by sharply delineated hyperechoic borders on either side of the hypoechoic tubular structure [75]. Nerves can be distinguished from tendons based on their echotexture, relative lack of anisotropy, anatomical location, and proximity to blood vessels [63,65,76].

#### 3.1.6. Arteries

Ultrasound imaging reveals veins and arteries as tubular structures that can appear either hypoechoic or anechoic. These vessels are easily compressible, and Doppler imaging effectively shows blood flow within them [20]. The hemodynamic parameters and flow patterns in the femoral artery of dogs have been studied, revealing a typical three-phase waveform [77,78].

### 3.2. Imaging of Pathological Musculoskeletal Structure

#### 3.2.1. Muscle

Ultrasound imaging can track the muscle from its origin to its insertion in a single scan, identifying trauma that may result in partial or complete disruption of muscle fibers [44,79]. Injuries are usually characterized by an irregular cavity, which frequently may contain a hematoma [44]. Furthermore, ultrasound examination is capable of differentiating between various pathologies and heterogeneous fluid collections, such as hemorrhage, abscesses, and tumors [79,80,81]. The characteristics of the muscle injuries differ based on their age and severity and are marked by a disruption in normal echogenicity [21]. Ruptured muscle fibers are recognized as hyperechoic scar formations, and the diameter of the injured muscle is reduced. In cases of muscle atrophy, the echogenicity of the affected muscles appears slightly elevated, with no signs of inhomogeneity [21].

#### 3.2.2. Tendon and Ligament

A partial rupture may lead to thinning of the tendon. In contrast, a complete rupture typically presents as a discontinuation of the tendon. This is often associated with changes indicative of tendinosis [16,63]. After a rupture, the tendon shows a significant increase in diameter compared to its original size. Initially, it exhibits hypoechogenicity, which later transitions to hyperechogenicity. As time progresses, the tendon diameter decreases, and the characteristic fibrillous structure starts to reappear [21]. Chronic tendon injuries may appear normal to hyperechoic, showing signs of narrowing, fiber reorganization, and possible dystrophic mineralization [18]. In human medicine, increased vascularity has been observed in Achilles tendon ruptures when assessed with CEUS. In contrast, power Doppler imaging fails to identify this vascular change [82,83].

#### 3.2.3. Bone

Early signs of osteomyelitis are characterized by the presence of hypoechoic fluid along the bone surface. As the condition progresses, the bone surface may become irregular; however, significant cortical disruption is typically absent [18,21]. In cases where the cortex is damaged, a thin hyperechoic line parallel to the cortical bone, known as a periosteal reaction, may be observed [62]. Additionally, bone fractures can also be diagnosed using ultrasound [47].

#### 3.2.4. Stifle Joint

OA is characterized by pathological changes, including loss of cartilage contour and thinning, accompanied by alterations in echogenicity [84]. This condition results in asymmetric narrowing of the cartilaginous layer, often associated with a hyperechoic signal at the joint attachment site [47,68,69]. OA lesions appear as hyperreflective areas with irregular borders on the bone surface, whereas osteochondrosis is characterized by focal cartilage defects. Additionally, free-floating cartilage fragments may be observed as hyperreflective foci within the joint fluid [12].

**Figure 2:**
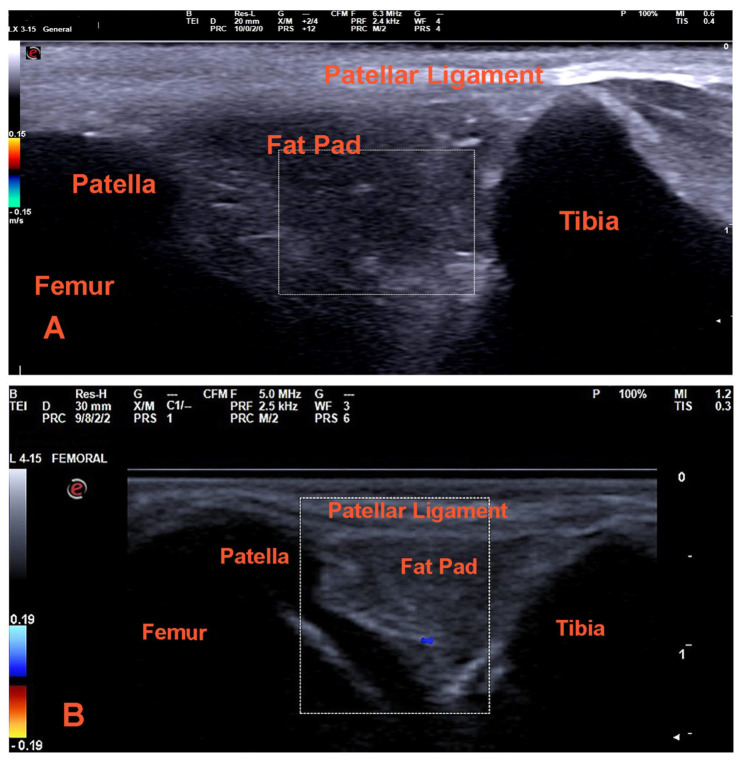
Color Doppler ultrasound images of the stifle joint. (**A**) Normal joint characterized by an absence of color signal, indicating no detectable flow; (**B**) stifle joint of a dog with patellar luxation, which shows detectable flow (blue dot). The images were obtained with a pulse repetition frequency (PRF) set at 2.4–2.5 MHz. (Ultrasound images: A. Karatrantos.)

In inflammatory conditions, the synovium typically appears thickened, hypertrophic, and edematous, manifesting as a hypoechoic band between muscle and fat [60,85]. Synovitis may be indicated by hyperemia detected through Doppler examination, and intra-articular fluid may be present [86,87,88] (Figure 1 and Figure 2). Assessment of synovial thickness serves as an effective method for evaluating the efficacy of treatment in inflammatory arthritis [89,90].

In animals with chronic joint disease, ultrasonographic findings may reveal either an excess or a deficiency of synovial fluid [47], potentially accompanied by pus, blood clots, fat lobules, or osteochondral fragments [91]. Even minimal joint effusions can be detected using ultrasonography [92] and aspiration may be necessary in these instances [60]. Osteophytes can be identified as hyperechoic, rough, and irregular formations [21,47,93,94]. While ultrasonography cannot directly visualize cartilage injuries within the joint, it can reveal secondary indicators suggestive of cartilage damage [44].

Neoplasia in the stifle is uncommon, with synovial cell sarcoma representing the most frequently encountered type. Definitive diagnosis requires a biopsy for histopathological confirmation [14,95]. Tumors typically appear as inhomogeneous areas with irregular borders, exhibiting echogenicity that ranges from hypoechoic to hyperechoic [47]. Synovial osteochondromas affecting the stifle are benign and generally present as well-defined, rounded masses containing multiple calcified intra-articular nodules [13,95].

- Cranial Cruciate Ligament (CCL)

The rupture of the CCL is the most common cause of stifle OA in dogs and is frequently associated with concomitant injury to the medial meniscus [96,97,98,99]. Ultrasonographic evaluation has proven an effective method for confirming CCL ruptures [48,70]. A recent study found that dogs with chronic CCL disease showed increased thickness and rigidity of the patellar ligament, as assessed using strain elastosonography [37]. The authors observed a significant difference in mechanical properties between healthy tendons and those affected by CCL rupture, with stiffness progressively increasing with the disease duration [37].

- Patellar Ligament

The patellar ligament may be observed as a thickened, hypoechoic area, which could suggest hypertrophy [100,101]. During ultrasound evaluation, a ruptured patellar ligament is typically characterized by swelling and irregular margins [6,47,73,102].

In SE assessments in healthy dogs, the patellar tendon is identified as an intermediate to soft structure, with increased stiffness levels in cases of CCL rupture [103].

- Other tendons and ligaments

Collateral ligament injuries appear hypoechoic to anechoic and exhibit echotextures that range from homogeneous to inhomogeneous [12]. Avulsion of the long digital extensor tendon is identified as a hyperreflective structure that displays acoustic shadowing [12].

- Meniscus

Ultrasonography can detect pathological conditions of the meniscus through various indicators. These include fluid accumulation around the meniscus and changes in echogenicity. Alterations in shape and displacement from the normal position are also significant. This is especially true in cases of a displaced bucket handle tear [49]. Meniscal lesions are typically imaged as heterogeneous areas with reduced echogenicity and are associated with meniscal swelling [48,91].

#### 3.2.5. Peripheral Nerve

Abnormal ultrasonographic findings in the branches of the sciatic nerves, namely, the common peroneal and tibial nerves, include either increased thickness, irregular borders, or both [75]. Additionally, color Doppler imaging can reveal an increase in blood flow within the nerve [104].

#### 3.2.6. Arteries

Hemodynamic parameters and flow patterns in the femoral artery have been studied in dogs [77,78]. Changes in the typical three-phase waveform can be observed in various conditions, including arterial pathologies, tumors [77], and systemic arterial dirofilariasis [105]. Additionally, power Doppler has been used in human medicine to assess treatment responses in patients with rheumatoid arthritis [106]. Alterations in blood flow are considered a key factor in the onset and progression of knee OA in humans [107,108]. Studies have shown that inadequate blood flow to the subchondral bone can disrupt nutrient diffusion to the articular cartilage in knee OA [109]. Furthermore, ischemia in the subchondral bone can lead to subsequent joint damage [110]. Boyaci et al. (2015) found that blood flow volume in the major arteries supplying the knee joint is significantly higher in patients with symptomatic knee OA compared to normal levels [111].

## 4. Other Imaging Modalities

In clinical practice, the diagnosis of stifle joint disorders generally depends on the history of lameness, the physical examination findings, and the radiographic imaging [6,12,95]. Pathological conditions like patellar luxation [13,14,100,112,113,114,115,116], early-stage infectious arthritis [13,95], and OA [12] can be detected radiographically. In some instances, radiographic stress views may be needed to validate the diagnosis of joint disease [13,95]. However, if the radiographic evaluation is inconclusive, more advanced imaging techniques may be required [12].

In human medicine, MRI and computed tomographic arthrography (CTA) are considered the gold standards for the non-invasive detection of meniscal lesions [117,118]. However, MR arthrograms are also used [119,120,121]. In dogs, MRI and CTA have also been proposed for the evaluation of ligaments and identification of intraarticular structures [122,123,124,125,126].

## 5. Discussion

Disorders of the stifle joint are a common cause of hindlimb lameness in dogs [127], with the CCL rupture being the most prevalent [128]. Ultrasonography serves as a valuable diagnostic tool for evaluating a wide range of musculoskeletal abnormalities, including cartilage lesions, meniscal tears, and pathologies involving muscles, tendons, ligaments, and nerves [12,44]. Specifically, it can identify patellar luxation, fractures [47], OA, osteochondrosis, damaged menisci, ligament injuries, and neoplasia [12]. Moreover, ultrasonography facilitates fine-needle aspirates and core biopsies, enabling accurate targeting of affected soft tissue structures [18].

Ultrasound and radiology work in tandem to assess both soft tissue and bone pathology [70]. Ultrasonography is not intended to replace radiography but rather to serve as a complementary modality [18]. This is particularly advantageous in situations where distinguishing between soft tissue presence or synovial effusion is essential, and radiography alone is insufficient [70]. Comparative studies have revealed the diagnostic benefits of ultrasonography over radiography in certain contexts. For example, Carr and coauthors [129] reported that ultrasound demonstrated superior efficacy in detecting patellar ligament pathologies, compared to radiographic examination. While ligament thickening could be identified radiographically during follow-up, differentiation between periligamentous and ligamentous thickening proved difficult in some dogs due to inadequate contrast. On the contrary, ultrasonography allowed for clear differentiation between tendon and periligamentous tissues [129]. Furthermore, Gnudi and Bertoni [70] demonstrated that ultrasonography could identify synovitis in 25% of dogs without any radiographic evidence of OA, thus enabling early diagnosis. In addition, among dogs with varying degrees of osteoarthritic changes on radiographs, ultrasonography provided valuable information regarding the chronicity and intensity of the inflammatory process in 70% of cases [70].

On the other hand, ultrasound and MRI imaging have been recognized as effective alternative diagnostic techniques to conventional radiology for examining the stifle joint, in both small and large animals [5,6,21,130,131,132,133,134,135]. Many studies have effectively utilized MRI to document and quantify OA in dogs, especially in naturally occurring and experimental cases of CCL deficiency [124,135,136,137,138,139]. OA is mainly assessed through radiography in both humans and animals [96,140,141,142,143]. Nevertheless, MRI has proven to be superior to radiography for detecting early OA in canine experimental models [144]. However, due to the high cost of MRI, ultrasound is preferred as a dynamic examination [145,146].

In human medicine, the sensitivity and specificity of B-mode ultrasonographic evaluation of meniscal lesions are found to be high [147]. The same applies to dogs [48,49], although it largely depends on the ultrasonographer’s experience and the quality of the equipment used [147,148,149,150]. Several factors may limit the effectiveness of ultrasonography for a thorough evaluation of the meniscus, including severe fibrosis of the soft tissues on the medial side of the joint, advanced OA with significant osteophytosis, and prior surgical interventions [49]. Typically, the medial meniscus is more easily assessed via ultrasonography, whereas the lateral meniscus can be more challenging to evaluate due to the narrower observation window between the lateral femoral condyle and the lateral tibial plateau [49].

Besides B-mode, other ultrasonographic methodologies contribute to the diagnostic procedure of stifle pathology, like the Doppler technique and CEUS. Many studies have confirmed that power Doppler imaging can detect neovascularization in the joint [151,152]. Power Doppler has been used in humans for assessing neovascularization in bone fractures [153,154]. The application of power Doppler for the evaluation of fracture healing in dogs and cats was described by Risselada et al. [26]. That study found a time-dependent development and then regression of vascularization of the uncomplicated fracture healing in long bones [26]. Furthermore, Pappa and colleagues conducted an ultrasonographic evaluation of the length of bone defects and vascularization in sheep during the bone healing process [155]. The power Doppler methodology was also employed to differentiate between infectious and noninfectious joint effusions in rabbits [27]. That study stated that an elevated power Doppler signal indicated a strong inflammatory response; however, the lack of an increased power Doppler signal did not exclude the possibility of infection, so aspiration was necessary for further evaluation [27]. Additionally, a hemodynamic parameter known as the pulsatility index (PI) has been identified as the most effective indicator of synovial vascularization in the knee joint when assessed using power Doppler [28]. A study conducted on a canine tibial osteotomy model investigated neovascularization during bone healing using contrast-enhanced ultrasonography and power Doppler imaging by Jeon et al. [156] revealed that vascular signals from the soft tissue were initially detected on day 2 by both techniques. However, CEUS identified a greater number of vascular signals compared to power Doppler on the same day [156]. CEUS was found to be more sensitive than power Doppler ultrasonography in detecting neovascularization in the surrounding soft tissue and associated callus [156]. CEUS functions as an objective and quantitative evaluation tool, facilitating the diagnosis of various musculoskeletal pathologies and predicting treatment outcomes [29]. It has shown effectiveness in differentiating the causes of fracture nonunions and in identifying the nature of tumors [29]. In humans, CEUS has also been used in the detection of synovitis in patients with knee OA [157]. A study in rabbits indicated that CEUS could detect synovial neovascularization during the early stages of rheumatoid arthritis [28]. CEUS is more sensitive than power Doppler in this initial phase of the disease, and its semiquantitative and quantitative analyses are especially valuable [28]. However, CEUS has certain limitations, including being invasive, costly, and time-consuming [28]. Additionally, it can only assess a single plane or cross-sectional perfusion of one joint at a time [28].

Another ultrasonographic modality is elastography. Tissue elasticity refers to its ability to return to its original shape after deformation due to stress [158]. These strain data can be illustrated through a color map or expressed as a quantitative ratio [158,159]. Elastography enables the qualitative and quantitative evaluation of the mechanical properties of tissues, focusing specifically on their stiffness and elasticity [160]. Elastography is an effective imaging technique for evaluating the softness/stiffness of the patellar ligament of dogs, without the need for sedation or a contrast medium [33]. Both elastography methodologies have been described for the assessment of the patellar ligament in healthy dogs [161]. In human medicine, SWE has been shown to be highly effective for the early detection of musculoskeletal diseases [39]. Additionally, patellar tendon stiffness measured by SWE has demonstrated a strong correlation with MRI findings in cases of human patellar tendinitis [162]. In dogs, it has been found that SWE can determine the elastic modulus in muscle; however, muscle stiffness varies with joint angle and becomes higher as the muscle is elongated [32]. Moreover, research on dogs has investigated the clinically healthy patellar tendon through SE [33,34], as well as after injury [37]. Another study assessed the feasibility of SWE on the normal canine common calcaneal tendon [35]. A new approach to elastography, the ARFI generates dynamic stress through mechanical shear waves, allowing for qualitative and quantitative evaluations of tissue elasticity [159,163]. ARFI elastography of the stifle joint of healthy beagles was published by Diogo et al. [36]. It is important to approach comparisons of results from different techniques of elastography with caution, as there are notable differences between methodologies.

Generally, joint disorders of dogs can be approached diagnostically by various methods, including physical examination, arthroscopy, radiography, ultrasonographic modalities, computed tomography (CT), and MRI [28,33,164,165,166,167]. Ultrasound and MRI are primarily used for the evaluation of the patellar ligament [161]. Both B-mode ultrasound and MRI have limitations in demonstrating the mechanical and functional properties of tissues [168], while elastosonography is a complementary imaging technique to ultrasound that assesses the mechanical properties of tissue [37]. In the field of veterinary practice, the cost of MRI and CT in comparison tο ultrasound is considered higher [15,145]. Thus, ultrasound is preferred as it can be used to accurately characterize the size, shape, and composition of menisci in dogs [169,170]. Earlier studies [6,70] indicated that the caudal half of each meniscus was not sufficiently assessable through ultrasonographic examination. However, Mahn et al. [49] demonstrated that both the lateral and medial menisci of dogs with an average weight could be effectively evaluated using ultrasonography, facilitating accurate pathology interpretation. Additionally, ultrasonographic modalities have been employed to evaluate the healing process of musculotendinous injuries through follow-up examinations [18].

In conclusion, a variety of ultrasound techniques should be utilized for the most effective assessment of knee joint pathology. The choice of ultrasound methods must be tailored to the specific structures being evaluated (such as tendons or menisci) and the type of information needed by the examiner (including tissue elasticity or neovascularization). For instance, power Doppler and CEUS can serve as supplementary examinations to assess neovascularization in the affected area [29] in cases of infection, fractures, or malignancy. Meanwhile, SWE is valuable for evaluating the stiffness of tendons and ligaments. B-mode imaging is regarded as a fundamental technique for visualizing anatomical structures, detecting ruptures, and identifying the presence of calcifications.

## 6. Conclusions and Future Directions

The stifle joint presents a diagnostic challenge due to its intricate anatomy. Although MRI has recently been recognized as the gold standard in human medicine, ultrasound offers several advantages in veterinary practice, including lower costs, greater availability, faster procedures, ease of repeat examinations, real-time assessments, and minimal need for sedation or general anesthesia. This does not indicate that ultrasound can replace other imaging techniques, such as CT or MRI.

B-mode ultrasound can detect bone fractures, masses, tendon and ligament ruptures or inflammation, joint effusion, thickening of the joint capsule, and cartilage defects. Additionally, joint instabilities are often evaluated through dynamic ultrasonographic examinations. Furthermore, ultrasound provides real-time guidance for safe and accurate needle aspiration. When B-mode imaging is combined with advanced techniques such as Doppler, CEUS, and elastography, diagnostic accuracy improves, aiding therapeutic decisions and enhancing clinical outcomes. While high-frequency ultrasound delivers excellent resolution, its tissue penetration ability decreases, creating a delicate balance. Consequently, deeper structures may be less clearly visualized due to the attenuation of ultrasound waves. Artifacts can arise from various sources, including patient movement and surrounding tissues. If these artifacts are not identified and addressed during analysis, they may lead to misinterpretation.

## Data Availability

Not applicable.
